# Supplementary material for: Pre-clinical study of induced pluripotent stem cell-derived dopaminergic progenitor cells for Parkinson’s disease
Source: Nat Commun. 2020 Jul 6;11:3369. doi: 10.1038/s41467-020-17165-w (PMC7338530; doi:10.1038/s41467-020-17165-w)
Supplement: Supplementary file 1 — Supplementary Information [file 41467_2020_17165_MOESM1_ESM.pdf]

# Supplementary Figure 1

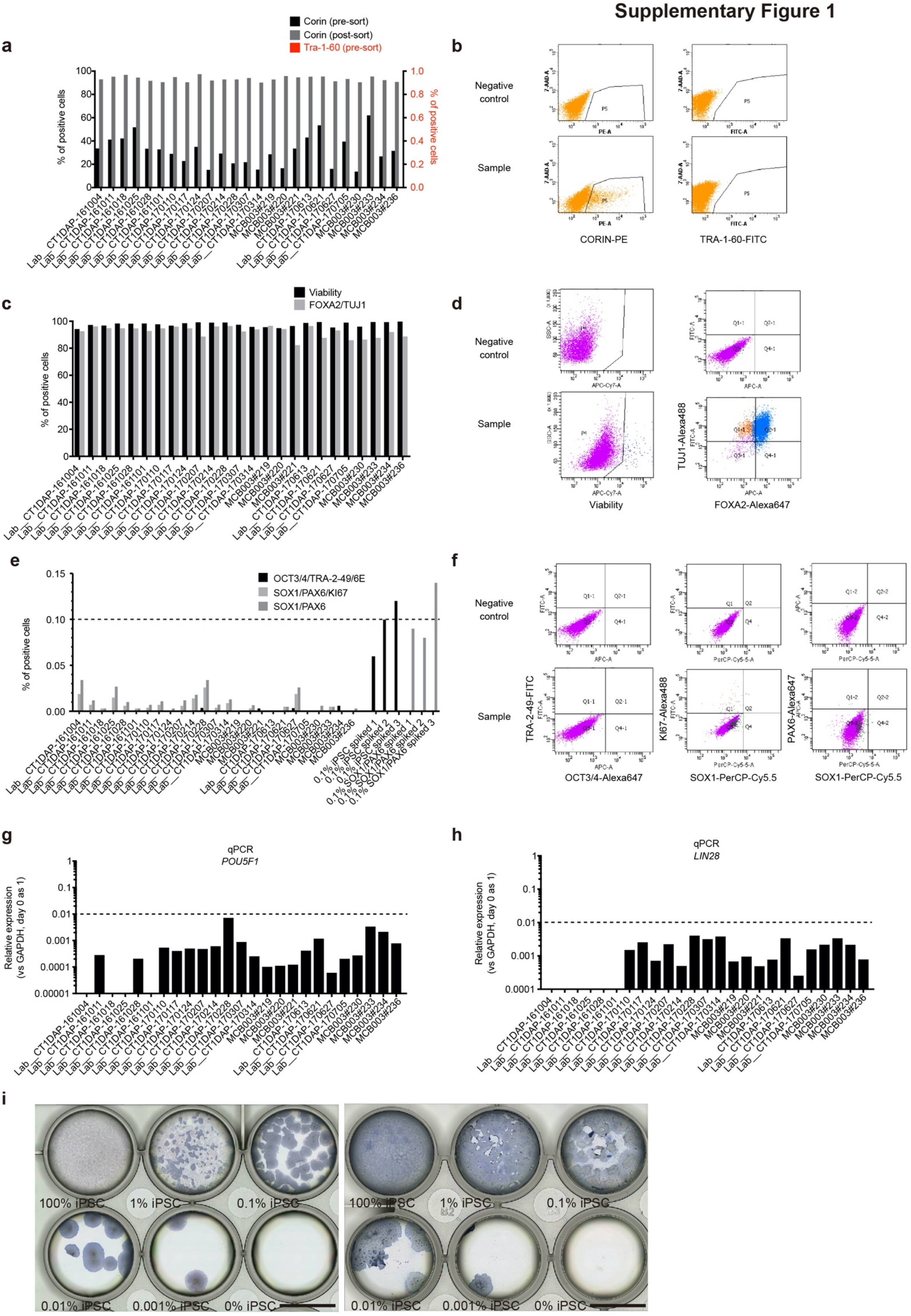

Supplementary Figure 1

a) Flowcytometry results of CORIN positive (left Y axis, pre-and post-sorting) cells and TRA-1-60 positive (right Y axis) cells in 25 lots of DAPs on culture day 12. b) Representative dot plot images exemplifying the gating strategy of flowcytometry, corresponding to Supplementary Figure 1a. The positive gate was set so that less than 0.1% of cells were positive in negative control sample. c) Flowcytometry results of cell viability and FOXA2 and TUJ1 (efficacy markers) positive cells in 25 lots of DAPs on culture day 26. d) Representative dot plot images exemplifying the gating strategy of flowcytometry, corresponding to Supplementary Figure 1c. The positive gate was set so that less than 0.1% of cells were positive in negative control sample. e) Flowcytometry results of OCT3/4, TRA-2-49/6E, SOX1, PAX6, and KI67 (safety marker) positive cells 25 lots in DAPs on culture day 26. f) Representative dot plot images exemplifying the gating strategy of flowcytometry, corresponding to Supplementary Figure 1e. The positive gate was set so that less than 0.1% of cells were positive in negative control sample. g, h) Gene expressions of *POU5F1* (g) and *LIN28* (h) by RT-qPCR analysis in 25 lots of DAPs on culture day 26. The expression level of undifferentiated iPSCs was set to 1. i) In vitro detection of residual undifferentiated iPSCs in iPSC maintenance condition. Several spiking iPSCs were mixed with dissociated DAPs and cultivated for 14 days. Colonies of undifferentiated iPSCs were stained by alkaline phosphatase. Cells in 100% iPSC condition formed too many colonies, resulting in spontaneous differentiation. Bars=1 cm (n=3 independent experiments).

## Supplementary Figure 2

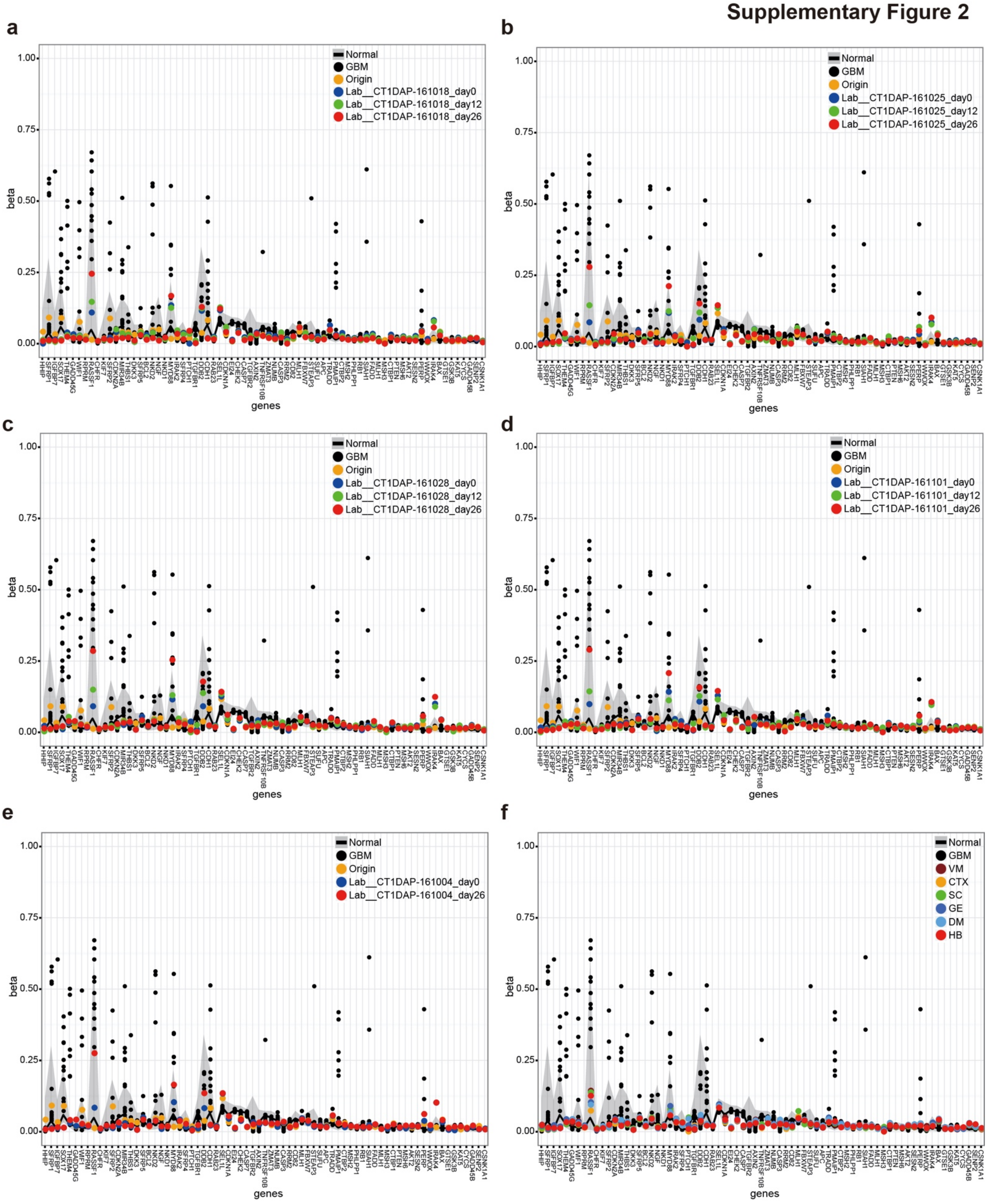


Supplementary Figure 2

(a-f) Results of the methylation array analysis of 73 cancer-related genes in 5 samples used in the tumorigenicity study (a-e) and human fetal tissue (f) as a control. GBM, glioblastoma multiforme; VM, ventral mesencephalon; CTX, cortex; SC, spinal cord; GE, ganglionic eminence; DM, dorsal mesencephalon; HB, hindbrain.

# Supplementary Figure 3

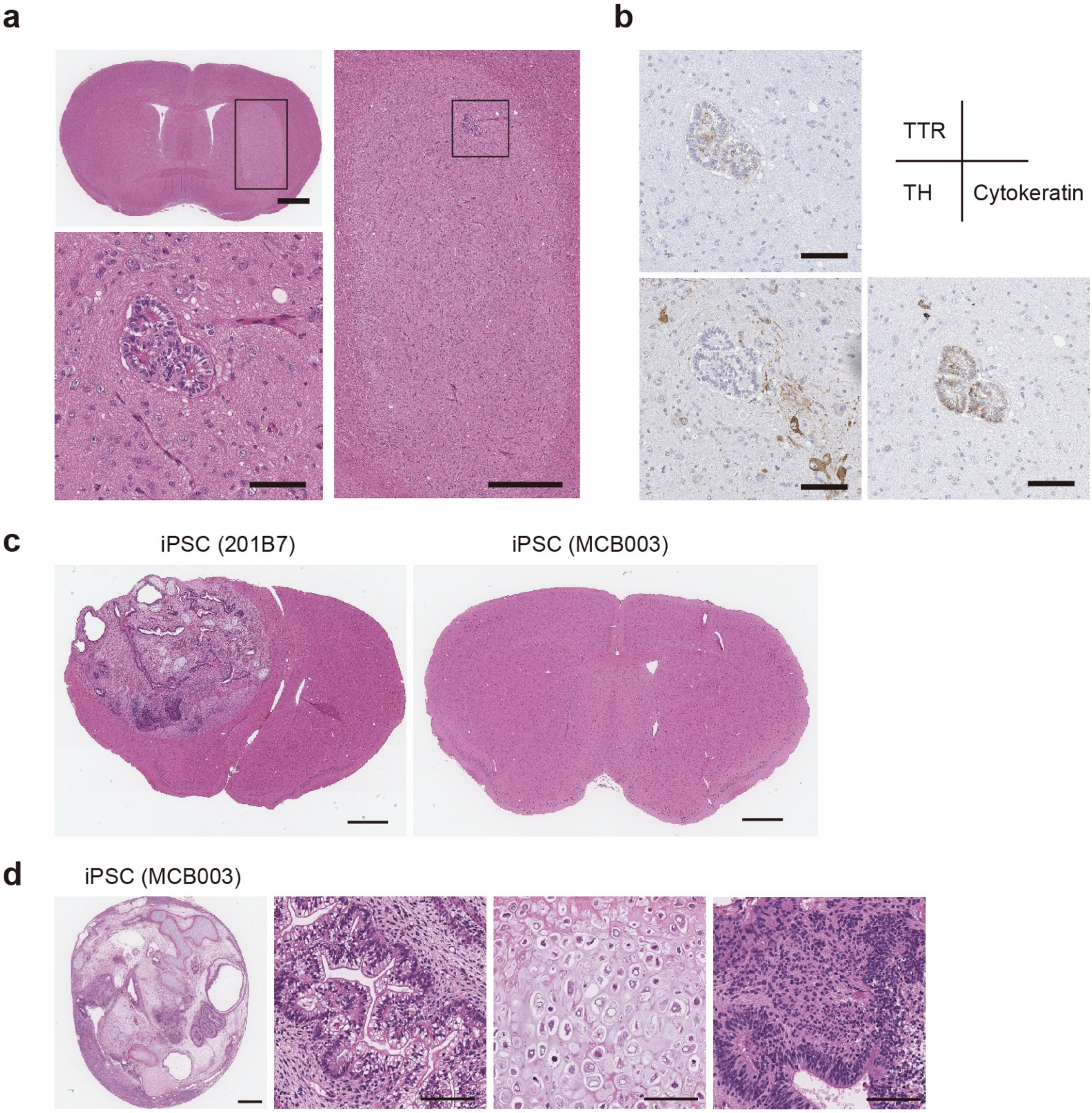


Supplementary Figure 3

a) Images of H-E staining of epithelium-like cell clusters found in the brain of NOG mice. Right is a magnification of upper left, and lower left is a magnification of right. Bars: left upper=1 mm, left lower=100 µm, and right=500 µm. b) Immunohistochemistry of the cell clusters. TTR, transthyretin; TH, tyrosine hydroxylase. Bars=100 µm. a-b) n=6 independent experiments. c) H-E staining of mouse brains injected with undifferentiated 201B7 (number of cell preparations=2 and number of animals=6) and MCB003 (number of cell preparations=2 and number of animals=7). Bars=1 mm. d) H-E staining of testis of NOG mice injected with undifferentiated MCB003 (number of cell preparations=1 and number of animals=6). Bar in leftmost panel=1 mm, others=100 µm.

## Supplementary Figure 4

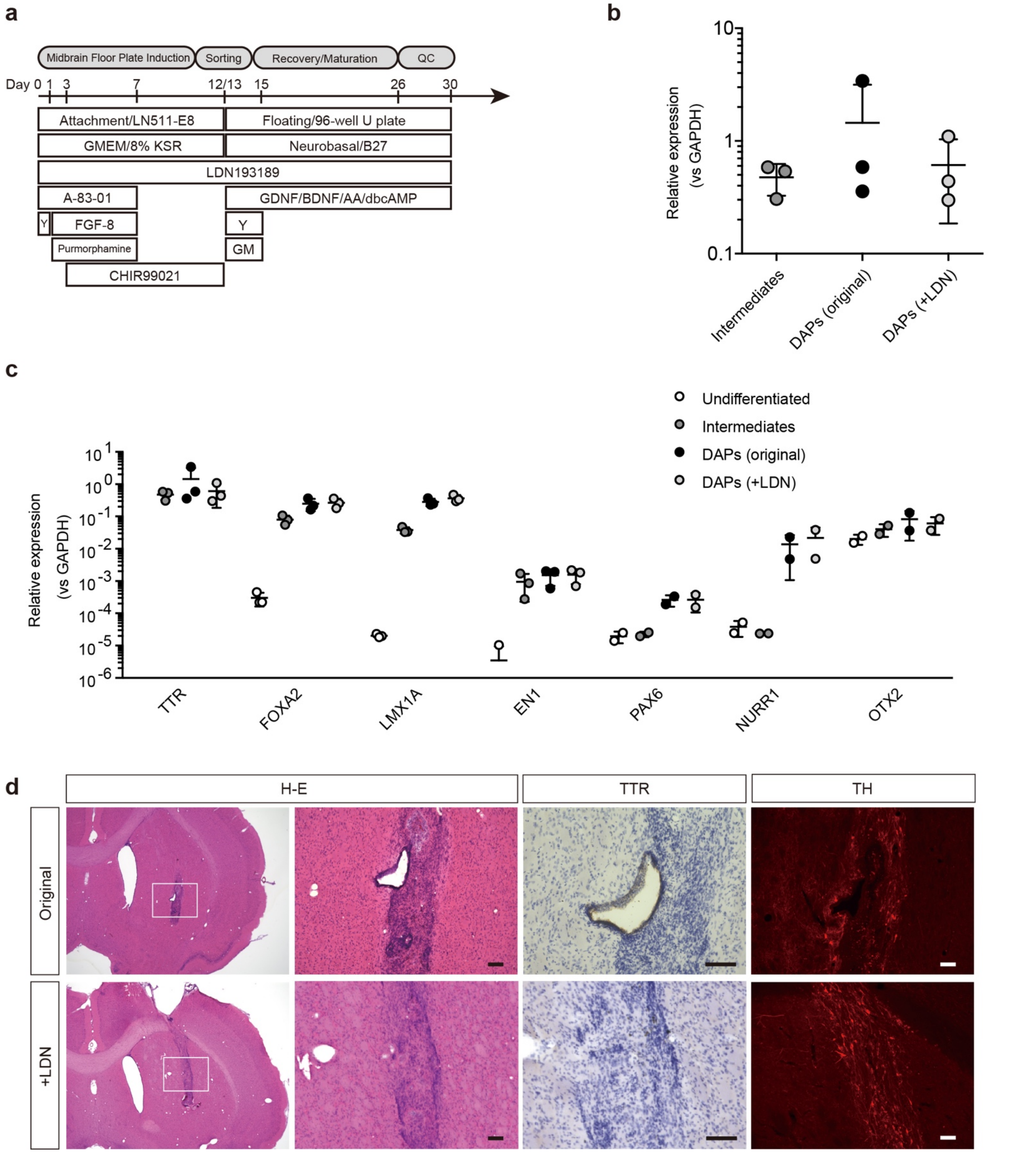


Supplementary Figure 4

a) The modified culture protocol (+LDN). b) RT-qPCR results of the expression of TTR in intermediates (day 12) and the final product (DAPs) in the original and modified culture protocols. Values are the mean±SD (n=3 independent experiments). c) Gene expression patterns of dopaminergic neuron-related genes by RT-qPCR. Values are the mean±SD (n=3 independent experiments). d) H-E staining and immunohistochemistry of cells injected into the brain of a nude rat with the original (number of animals=7) or modified culture protocol (number of animals=6). Left images are magnified in the other images. Bars=100 μm. Number of cell

preparations=3.

Supplementary Table 1. Modifications between laboratory-grade and GMP-grade cell production

| | **Laboratory Use (ref. 8)** | **Pre-clinical / Clinical trial** |
|---|---|---|
| Sorting Buffer | PBS (-) or HBSS (-) | PBS (-) |
| | 2% FBS | 2% FBS, gamma-ray irradiated |
| | 50 µg/mL Penicillin/Streptomycin | 80 µg/mL Gentamycin |
| Cell Dissociation Enzyme | 1×Accumax | 0.5×TrypLE Select (with 0.5 mM EDTA/PBS (-)) |
| Dead Cell Exclusion | 7-AAD | None |
| Anti-CORIN Antibody | Gift from KAN Research Institute | Phycoerythrin-conjugated antibody (Sumitomo Dainippon Pharma) |
| Negative control | Stained with 2nd antibody | Unstained sample or stained with PE-isotype control |
| Differentiation media | KSR | Gamma-ray irradiated KSR |
| | Neurobasal/B27 w/o vitamin A | Neurobasal/B27 w/o vitamin A (gamma-ray irradiated B27) |
| | 2 mM L-glutamate | 2 mM Glutamax |
| | 50 µg/mL Penicillin/Streptomycin | 80 µg/mL Gentamycin |
| Replating plate | Lipidure-coated 96-well plate (U-shaped) | PrimeSurface 96U plate (Sumitomo Bakelite) |

Supplementary Table 2. Characterization of the human iPSC master cell bank (MCB003)

| **Test Item** | **Test Method** | **Criteria** | **Results** |
|---|---|---|---|
| Morphology | Microscopy observation | Human iPSC-like morphology | Complied |
| Cell count | Cell count | For information only | $3.1\times10^{6}$ cells/tube |
| Viability | Cell count | For information only | 93% |
| Growth Rate-Doubling | Cell count | For information only | 14.6 hours |
| Undifferentiated markers | Flowcytometry | TRA-1-60 ≥ 90 % | 97.6 % |
| | | TRA-2-49 ≥ 90 % | 100 % |
| | | SSEA4 ≥ 90 % | 100 % |
| Sterility | JP17[a] (direct method) | Complies | Complied |
| Mycoplasma | JP17 (nucleic acid amplification test) | Negative | Negative |
| Endotoxin | JP17 (LAL[b] turbidimetry test) | < 24 EU/mL | < 0.05 EU/mL |
| Karyotype | G-band | Normal | Normal |
| Viral testing | ICH Q5A | Negative | Complied |

[a]The Japanese Pharmacopoeia Seventeenth Edition

[b]Limulous amoebocyte lysate

Supplementary Table 3. In-process testing for the manufacturing of DAPs

| **Process** | | **Test Items** | **Test Method** | **Criteria** |
|---|---|---|---|---|
| Prepared reagents (In-process test 1–6) | | Sterility | Membrane filter | Negative |
| Expand iPSCs (In-process test 1–3) | | Cell number | Cell counter | Required cell number |
| | | Morphology | Microscopy | Colony formation |
| | | Sterility | BacT/ALERT (Culture supernatant) | Negative |
| Differentiation | In-process test 5 | Cell number | Cell counter | Required cell number |
| | In-process test 4–6 | Morphology | Microscopy | Attached to the plate (test 3–5)<br>Sphere formation (test 6) |
| | | Sterility | BacT/ALERT | Negative |
| | In-process test 4, 6 | Mycoplasma | qPCR | Negative |
| | In-process test 6 | Endotoxin | LAL[a] turbidimetry test | ≤ 1 EU/mL |
| | (In-process test 5, pre-sort) | Residual undifferentiated iPSCs | Flowcytometry: TRA-1-60 | < 1% |
| | (In-process test 5, post-sort) | Purity of sorted cells | Flowcytometry: CORIN | > 90% |

[a] Limulus Amebocyte Lysate

Supplementary Table 4. Shibata's gene list proposed by the PMDA

| | | | | | | |
|---|---|---|---|---|---|---|
| ABL1 | CBFA2T3 | ERCC4 | GATA1 | MEN1 | NUP214 | SH3GL1 |
| ABL2 | CBLB | ERCC5 | GATA3 | MET | NUP98 | SMAD4 |
| ACVR1B | CBLC | ERCC6 | GNA11 | MITF | PALB2 | SMARCA4 |
| AFF3 | CCND1 | ETV4 | GNAQ | MLH1 | PAX8 | SMARCB1 |
| AKAP9 | CCND2 | ETV6 | GNAS | MLH3 | PBRM1 | SMO |
| AKT1 | CCND3 | EVI1 | GOLGA5 | MLL | PDE4DIP | SOCS1 |
| AKT2 | CDC73 | EWSR1 | GOPC | MLL2 | PDGFB | SRGAP3 |
| ALK | CDH1 | EXT1 | GPC3 | MLL3 | PDGFRA | SRSF2 |
| APC | CDH11 | EXT2 | H3F3A | MLLT3 | PDGFRB | SS18 |
| ARHGEF12 | CDK6 | EZH2 | HMGA1 | MPL | PIK3CA | STAT3 |
| ARID1A | CDKN2A | FAM123B | HMGA2 | MSH2 | PIK3R1 | STK11 |
| ARID2 | CDKN2C | FANCA | HNF1A | MSH6 | PIM1 | SUFU |
| ASXL1 | CDX2 | FANCB | HRAS | MUTYH | PLAG1 | SUZ12 |
| ATF1 | CEBPA | FANCC | IDH1 | MYB | PML | SYK |
| ATM | CHEK1 | FANCD2 | IDH2 | MYC | PMS2 | TCF3 |
| ATR | CHEK2 | FANCE | IKZF1 | MYCL1 | POLE | TCL1A |
| ATRX | CIC | FANCF | IL2 | MYCN | POLH | TET2 |
| AXIN1 | COL1A1 | FANCG | IL7R | MYD88 | PPARG | TFG |
| AXIN2 | CREB1 | FANCI | IRF4 | MYST3 | PPP2R1A | TLX1 |
| BAP1 | CREBBP | FANCJ | JAK2 | NCOA2 | PRKAR1A | TNFAIP3 |
| BCL11A | CTNNB1 | FANCL | JUN | NCOA4 | PTCH1 | TP53 |
| BCL11B | CYLD | FANCM | KDM5C | NF1 | PTEN | TPR |
| BCL2 | DAXX | FANCP | KDM6A | NF2 | PTPN11 | TSC1 |
| BCL3 | DDB2 | FBXW7 | KDR | NFE2L2 | RAD51C | TSC2 |
| BCL6 | DDIT3 | FEV | KIT | NFKB2 | RAF1 | TSHR |
| BCOR | DDX5 | FGFR1 | KRAS | NIN | RB1 | USP6 |
| BCR | DDX6 | FGFR1OP | LCK | NONO | REL | VHL |
| BHD | DEK | FGFR2 | LMO2 | NOTCH1 | RET | WRN |
| BLM | DICER | FGFR3 | MAF | NOTCH2 | RNF213 | WT1 |
| BMPR1A | DNMT3A | FH | MAFB | NPM1 | ROS1 | XPA |
| BRAF | EGFR | FLCN | MAML2 | NR4A3 | RUNX1 | XPC |
| BRCA1 | ELK4 | FLT3 | MAP2K4 | NRAS | SDHB | ZNF521 |
| BRCA2 | EP300 | FOXL2 | MDM2 | NSD1 | SDHD | |
| CARD11 | ERBB2 | FOXP1 | MDM4 | NTRK1 | SETD2 | |
| CARS | ERCC3 | FUS | MED12 | NTRK3 | SF3B1 | |

Supplementary Table 5. The results of the amplicon sequencing

| | | | | | | | Origin (PBMNC*) | | day0 | | day12 | | day26 | |
|---|---|---|---|---|---|---|---|---|---|---|---|---|---|---|
| Preparation | Chr** | Start | End | Ref | Alt | Gene name | coverage | alt_ratio*** | coverage | alt_ratio | coverage | alt_ratio | coverage | alt_ratio |
| CT1DAP-161025 | chr2 | 215657150 | 215657150 | T | A | BARD1 | 69 | 13.00% | 71 | 14.10% | 68 | 20.60% | 88 | 9.10% |
| CT1DAP-161028 | chr3 | 195512734 | 195512734 | T | G | MUC4 | 35 | 17.10% | 29 | 24.10% | 29 | 3.50% | 28 | 14.30% |
| CT1DAP-161101 | chr3 | 48719156 | 48719156 | C | T | NCKIPSD | 9911 | 0.10% | 19609 | 0.20% | 19451 | 0.10% | 12491 | 0.10% |

*Peripheral blood mononuclear cells

**Chromosome

***alteration ratio

Supplementary Table 6. Primer list of the amplicon sequencing

| Gene_ID | sequence_l | pos_l | sequence_r | pos_r | size |
|---|---|---|---|---|---|
| BARD1 | CCGGCTTGGACAACATAGAGAGACTC | 215656838 | GGAATTAAATTCTGCTGAATGGGTTGC | 215657225 | 388 |
| MUC4 | GTGTCACCTGTGGATGCTGAGGAAA | 195512650 | GCATCTTCAGGTCACACCACCTCTC | 195512906 | 257 |
| NCKIPSD | CTTCTTCTTCTGCAGGGGGTTCAGG | 48718986 | TGGGCTGTGAAGGGTATGGTTTTCC | 48719278 | 293 |

Supplementary Table 7. Animal numbers used in the tumorigenicity study

| **Differentiation number** | **Animal number** | | | |
|---|---|---|---|---|
| | Control group | | Sample group | |
| | Male | Female | Male | Female |
| CT1DAP-161004 | 5 | 5 | 2 | 2 |
| CT1DAP-161011 | 5 | 5 | 12 | 12 |
| CT1DAP-161018 | 5 | 5 | 7 | 7 |
| CT1DAP-161025 | 5 | 5 | 8 | 8 |
| CT1DAP-161028 | 5 | 5 | 6 | 6 |
| CT1DAP-161101 | 0 | 0 | 5 | 5 |

Supplementary Table 8. Summary of pre-clinical *in vivo* studies of human iPSC-derived dopaminergic progenitors

| Study Aim | Host, Administration route | Duration | Number of animals | Number of cell preparation | Cells/ animal | Evaluation | Findings |
|---|---|---|---|---|---|---|---|
| Tumorigenicity Biodistribution Toxicology | NOG mouse, unlesioned, Striatum | Life-long (52 weeks) | N=80; DAPs | 6 | $2 \times 10^5$ | Mortality/Morbidity, General health, Neurobehavioral tests, Hematology/Blood chemistry, Urinalysis, Necropsy, Histopathology | No effect on mortality/morbidity, animal observations or pathology. No graft overgrowth. Asymptomatic ectopic structures (choroid plexus epithelium-like cells) observed within graft. Little proliferative capacity (scattered Ki67+ cells, negative in ectopic structures). |
| | | | n=50; Control (Saline) | | | | |
| Detection of residual iPSCs | NOG mouse, unlesioned, Subcutaneous with Matrigel | 26 weeks | n=20; DAPs | 7 | $6 \times 10^5$ | Teratoma formation Tumor size Histology | No teratoma formation in every DAP group. Teratoma formation was observed in the positive control group (other iPS cell line and HeLa cells). |
| | | | n=10; 100% iPSCs | | | | |
| | | | n=50; spiked with 10%~0.001% iPSCs | | | | |
| | | | n=10; iPSC (201B7) | | | | |
| | | | n=10; HeLa cells | | | | |
| | | | n=10; Control (Matrigel) | | | | |
| Efficacy | Nude rat, 6-OHDA lesioned, Striatum | 20 weeks | n=8; DAPs | 2 | $4 \times 10^5$ | Behavioral test Histology | Methamphetamine-induced rotational behavior improved 16 weeks after transplantation. Human-derived dopaminergic neurons survived. |
| | | | n=6; Control (Saline) | | | | |
| | Cynomolgus monkey, MPTP-lesioned, Putamen | 8-24 weeks | n=3; DAPs | 2 | $1.5–2.0 \times 10^6$ | Radiology (MRI) Histology | Grafts were detected in MRI images. Human-derived dopaminergic neurons survived. No ectopic structures of dopaminergic neurons survived in the graft. |

Supplementary Table 9. 2 × 2 matrix for quality control of cellular products

| | **SAFETY** | | **EFFICACY** | |
|---|---|---|---|---|
| **CELLULAR COMPONENTS** | Unwanted cells | Undifferentiated iPSCs<br>Early neural stem cells<br>Transformed cells | Active cells | DA progenitor cells |
| **NON-CELLULAR COMPONENTS** | Process-derived impurities | Animal-derived materials<br>Anti-CORIN antibody | Active ingredients | Dopamine |
| | Adventitious microbial agents | Bacteria<br>Mycoplasma<br>Endotoxin<br>Virus | | |

Supplementary Table 10. PMDA requirements and solutions in this study

| Issues | | PMDA requirement | Solutions |
|---|---|---|---|
| Quality of cell product | Reagents | Meet Japanese standards of biological raw materials | Complied with requirement |
| | Residual iPSCs in final product | Assess the result of tumorigenicity study | Set criteria as OCT3/4/TRA-2-49/6E: <0.1% in flowcytometry and confirm no tumor formation |
| | Residual immature neural cells | Sox1/PAX6 positive cells should be evaluated | Set criteria as SOX1/PAX6: <0.1% in flowcytometry and confirm no tumor formation |
| | Residual vectors | To be confirmed in iPSCs | Confirm no residual plasmids in iPSCs and final product |
| | Genome/epigenome abnormality | No further request | WGS, SNP array / Methylation array |
| | Non-cell impurities | <120 μg/day (ICH M7) | Complied with requirement |
| Tumorigenicity/ Toxicity/ Biodistribution | Host species | NOG mice | NOG mouse / MPTP monkey |
| | Animal number per group | At least 10 animals for histological evaluation | Started 40 animals per group |
| | Observational period | Life-long (as long as possible) | Life-long (52 weeks after transplantation) |
| | Injection site | Same as clinical cases | Brain (striatum) |
| | Teratoma formation | Subcutaneous space of NOG mice, with Matrigel | Subcutaneous injection with Matrigel<br>Testis injection |
| Efficacy study | | No further request | 6-OHDA nude rat / MPTP monkey |

Supplementary Table 11. Assessment of viruses in the iPSC-derived master cell bank

| **Test Items** | **Test Method** | **Results** |
|---|---|---|
| Infectivity | Co-culture with HEK293 | Retrovirus was not detected |
| Electron Microscopic Observation | Electron microscopic observation | No extraneous agents observed in the 200 cell profiles examined |
| Reverse Transcriptase Activity | PCR (Polymerase chain reaction) | Pass |
| *In vitro* Assays | 3 detector cell lines: MRC-5, Vero, and HeLa cells | Pass |
| *In vivo* Assays | Suckling mouse, adult mouse, guinea pigs and embryonated eggs | Pass |
| Cell Specific Virus Tests | PCR Target viruses:<br>HSV 1/2 (Herpes Simplex Virus Type 1 & 2)<br>B19 (Parvovirus B19)<br>EBV (Epstein-Barr Virus)<br>SV40 (Simian Virus 40)<br>hCMV / HHV5 (Human Cytomegalovirus / Human Herpesvirus Type 5)<br>HHV6 (Human Herpesvirus Type 6)<br>HHV7 (Human Herpesvirus Type 7)<br>HHV8 (Human Herpesvirus Type 8)<br>HTLV (Human T Cell Leukemia Virus)<br>HCV (Hepatitis C Virus)<br>HAV (Hepatitis A Virus)<br>HBV (Hepatitis B Virus)<br>HIV-I (Human Immunodeficiency Virus Type 1)<br>HIV-II (Human Immunodeficiency Virus Type 2)<br>HBoV (Human Bocavirus)<br>hMPV (Human metapneumovirus) lineage A and B<br>WNV (West Nile Virus)<br>VZV (VariCella Zoster Virus)<br>HuPyV (Human Polyoma Virus)<br>WUPyV (WU polyomavirus)<br>KIPyV (KI polyomavirus)<br>HPyV (Human polyomavirus) pathogen detection (MCPyV, HPyV6, HPyV7, TSPyV, HPyV9) | Negative |

Supplementary Table 12: Materials and reagents used in this study

| Product Name | Supplier | Product # | Specification |
|---|---|---|---|
| StemFit® AK03N Liquid A | Ajinomoto | – | 400 mL |
| StemFit® AK03N Liquid B | | – | 100 mL |
| StemFit® AK03N Liquid C | | – | 2 mL |
| OTSUKA NORMAL SALINE | Otsuka Pharmaceutical | 613 | 50 mL |
| OTSUKA DISTILLED WATER | | 1751 | 100 mL |
| ACTOSIN For injection (dibutyryl cyclic AMP) | Daiichi-Sankyo | – | 300 mg |
| PE-anti CORIN antibody | Sumitomo Dainippon Pharma | – | 1.0 mg/mL |
| Gentacin® 40 | Takata Pharmaceutical | – | 40 mg/1 mL |
| Ascorbic Acid | TOWA Pharmaceutical | – | 500 mg |
| CultureSure® 10mmol/l Y-27632 solution, animal-derived free | FUJIFILM Wako Pure Chemical Corporation | 039-24591 | 300 µL |
| CultureSure® 10mmol/l CHIR99021 DMSO Solution, Animal-derived-free | | 038-24681 | 300 µL |
| CultureSure® A-83-01 | | 039-24111 | 2 mg |
| CultureSure® CHIR99021 | | 034-23103 | 5 mg |
| CultureSure® DMSO | | 031-24051 | 10 mL |
| StemSure® 10mmol/l 2-mercaptoethanol solution (×100) | | 198-15781 | 100 mL |
| FGF8, human, recombinant, Animal-derived-free | | 067-06231 | 25 µg |
| Purmorphamine | | 166-23991 | 5 mg |
| BDNF, human, recombinant, Animal-derived-free | | 028-16451 | 10 µg |
| GDNF, human, recombinant, Animal-derived-free | | 070-06261 | 10 µg |
| 0.5mol/L-EDTA (pH8.0) | nakalai tesque | 06894-14 | 100 mL |
| D-PBS(-) without Ca and Mg | nakalai tesque | 14249-24 | 500 mL |
| iMatrix-511MG | Nippi | 892005 | 175 µg |
| BD sheath bag with DPBS and Filter | BD | 660592 | 4.5 L |
| Accudrop Beads CTT | BD | 625059 | 2.5 mL |
| Fetal Bovine Serum Gamma Irradiated by SER-TAIN™ Process Sourced in Australia | SAFC | 12007C | 500 mL |
| D-glucose solution, 45% in H2O, sterile-filtered, BioXtra, suitable for cell culture | SIGMA-ALDRICH® | G8769 | 100 mL |
| Sodium Pyruvate, 100 mM, sterile-filtered, BioReagent, suitable for cell culture | SIGMA-ALDRICH® | S8636 | 100 mL |
| Stemolecule™ LDN-193189 | REPROCELL | 04-0074 | 2 mg |
| B27 supplement (50×) without vitamin A | Thermo Fisher Scientific | 12587 | 10 mL |
| Cell Therapy Systems TrypLE™ Select CTS™ | | A12859 | 100 mL |
| Cell Therapy Systems GlutaMAX™- I CTS™ (100×)200mM | | A12860 | 100 mL |
| GMEM Glasgow Minimum Essential Medium (1×), liquid + L-Glutamine-TPB | | 11710 | 500 mL |
| KNOCKOUT™ SR Serum Replacment for ESCs/iPSCs (KSR) | | 10828 | 500 mL |
| MEM Non-Essential Amino Acids Solution (100×) | | 11140 | 100 mL |
| Neurobasal® medium (1×), liquid -L-Glutamine | | 21103 | 100 mL |
| Petri Dish for Cell/Tissue Culture 90φ | Sumitomo Bakelite | MS-13900S | 90 (φ) × 20 (H) mm |
| | | | Culture area: 57 $cm^2$ |
| MultiWell Plate for Cell/Tissue Culture 6F with lid | | MS-80060S | culture area: 9.2 $cm^2$ |
| | | | well volume: 16 mL |
| PrimeSurface®Plate 96U | | MC-9096UI | U-shaped bottom |
| | | | well volume: 0.3 mL |
| 15 mL Centrifuge Tube | | MS-56150S | 19 (φ) × 118 (L) mm |
| 50 mL Centrifuge Tube | | MS-56500S | 30 (φ) × 115 (L) mm |
| 2 mL Pipette | | MS-66020S | Volume: 2 mL |
| | | | Scale: 0.05 mL |
| 5 mL Pipette | | MS-66052S | Volume: 5 mL |
| | | | Scale: 0.1 mL |
| 10 mL Pipette | | MS-66102S | Volume: 10 mL |
| | | | Scale: 0.1 mL |
| 25 mL Pipette | | MS-66252S | Volume: 25 mL |
| | | | Scale: 0.2 mL |
| 50 mL Pipette | | MS-66500S | Volume: 50 mL |
| | | | Scale: 2 mL |
| Cell Scraper S | | MS-93101S | 10 mm width, 160 mm length |
| 0.5 mL Slim Tube | | MS-4701WS | 10.5 (φ) × 46 (L) mm |
| Reagent Reservoir | | MS-62803 | - |
| Lock Stand | | MS-78000 | 5/case |
| BOTTLE, 250ML,.45MM,PS,W/CAP,S | Corning | 430281 | 2/Bag, 24/Case |
| BOTTLE, 500ML,.45MM,PS,W/CAP,S | | 430282 | 2/Bag, 24/Case |
| ep Dualfilter T.I.P.S® 2 – 20 µL | Eppendorf | 0030 077.539 | 10 × 96 Eppendorf Tips |
| ep Dualfilter T.I.P.S® 2 – 200 µL | | 0030 077.555 | 10 × 96 Eppendorf Tips |
| ep Dualfilter T.I.P.S® 50 – 1000 µL | | 0030 077.571 | 10 × 96 Eppendorf Tips |
| ep Dualfilter T.I.P.S® 0.1 – 5 mL | | 0030 077.580 | 5 × 24 Eppendorf Tips |
| 1.5 mL tube | WATSON | T119-7155CS | 100/case |
| Nunc CryoTube 1.0 mL, Starfoot, External Thread Writing area | ThermoFisher Scientific | 375353 | 50/BAG, 500/CASE |
| Tube 5mL 12x75mm RBtm PP 500cs | Corning | 352063 | BAG: 25, CASE: 500 |
| Tube 5mL 12x75mm RBtm w/Strain | | 352235 | BAG: 25, CASE: 500 |
| Kit BD Cytopeia Fluidic 100 um Bag Ster1 | BD Biosciences | 660535 | 1kit/box |
| Assy Waste Line Extension 76 in SVC | BD | 661183 | – |
| O-RING VITON FOR PRESSURE VESSELS | | 645266 | 1 EA |
| Conn 4 Shutoff Male 1/16in | | 649677 | 0 EA |
| Conn 4 Shutoff Female 1/16in | | 649676 | 0 EA |
| Cell container (inner tube) | JMS | Custom | PET/PE film |
| Cell container (outer tube) | | Custom | |
| Sterile Acrodisc® Syringe Filters with Supor® Membrane | Pall Life Sciences | 4612 | 50 /PK |

Supplementary Table 13. Antibody list

| **Antigen** | **Host species** | **Supplier** | **Cat No.** | **Applications** | **Dilution** |
|---|---|---|---|---|---|
| LMX1A | Rabbit | Millipore | AB10533 | IF | 1:2,000 |
| FOXA2 | Goat | R&D | AF2400 | IF | 1:500 |
| NURR1 | Rat | Gifted from KAN laboratory | – | IF,IHC | 1:1,000 |
| TH | Rabbit | Millipore | AB152 | IF,IHC | 1:400 |
| | | | | DAB | 1:5,000 |
| OCT3/4 | Mouse | Santa Cruz Biotechnology | sc-5279 | IF | 1:200 |
| NANOG | Goat | R&D | AF1997 | IF | 1:500 |
| SOX1 | Goat | R&D | AF3369 | IF,IHC | 1:100 |
| KI67 | Rabbit | Novocastra | NCLKi67p | IF,IHC | 1:1,000 |
| KU80 | Rabbit | Cell Signaling | 2180 | IHC | 1:200 |
| PAX6 | Mouse | BD Pharmingen | 561462 | IF | 1:500 |
| TTR | Rabbit | Dako | A0002 | IF, DAB | 1:1,000 |
| | | abcam | ab92469 | IF, DAB | 1:250 |
| HNA | Mouse | Millipore | MAB1281 | IF | 1:400 |
| Pan-Cytokeratin (AE1/AE3) | Mouse | Dako | IS053 | DAB | 1 Drop |
| GFAP | Rabbit | Dako | Z0334 | IHC | 1:200 |
| **Secondary antibodies** | | | | | |
| Anti-Mouse IgG (H+L) Highly Cross-Adsorbed Secondary Antibody, Alexa Fluor 488 | Donkey | ThermoFisher | A21202 | IF | 1:400 |
| Anti-Rat IgG (H+L) Highly Cross-Adsorbed Secondary Antibody, Alexa Fluor 488 | Donkey | ThermoFisher | A21208 | IF | 1:400 |
| Anti-Goat IgG (H+L) Cross-Adsorbed Secondary Antibody, Alexa Fluor 488 | Donkey | ThermoFisher | A11055 | IF | 1:400 |
| Anti-Rabbit IgG (H+L) Highly Cross-Adsorbed Secondary Antibody, Alexa Fluor 488 | Donkey | ThermoFisher | A21206 | IF | 1:400 |
| Anti-Rabbit IgG (H+L) Highly Cross-Adsorbed Secondary Antibody, Alexa Fluor 594 | Donkey | ThermoFisher | A21207 | IF | 1:400 |
| Anti-Goat IgG (H+L) Cross-Adsorbed Secondary Antibody, Alexa Fluor 594 | Donkey | ThermoFisher | A11058 | IF | 1:400 |
| Anti-Mouse IgG (H+L) Highly Cross-Adsorbed Secondary Antibody, Alexa Fluor 647 | Donkey | ThermoFisher | A31571 | IF | 1:400 |
| **Conjugated antibodies** | | | | | |
| FITC-conjugated TRA-1-60 | Mouse | BD | 560380 | FCM | 1:10 |
| PE-conjugated CORIN | Mouse | Sumitomo Dainippon Pharma | – | FCM | 1:30,000 |
| Alexa488-conjugated TUJ1 | Mouse | BD | 560381 | FCM | 1:5 |
| Alexa488-conjugated KI67 | Mouse | BD | 561165 | FCM | 1:20 |
| Alexa647-conjugated OCT4 | Mouse | BD | 560329 | FCM | 1:5 |
| Alexa647-conjugated PAX6 | Mouse | BD | 562249 | FCM | 1:20 |
| PerCP-Cy5.5-conjugated SOX1 | Mouse | BD | 561549 | FCM | 1:20 |
| FITC-conjugated TRA-2-49 | Mouse | Millipore | FCMAB133F | FCM | 1:20 |

Supplementary Table 14. DNA Primer List

| Gene name | Primer Fw | Gene name | Primer Rv |
|---|---|---|---|
| AADC_Hu_Fw | AGCCCCTACTTCTTCGCCTA | AADC_Hu_Rv | GAGCCAGTCCATCATCACAG |
| ALCAM_Hu_Fw | CCGTGTCATGCACAATATCTGC | ALCAM_Hu_Rv | CTTTAGTCCTTCAACCTCCTGC |
| ALDH1A1_Hu_Fw | ATGCTTCCGAGAGGGGGCGA | ALDH1A1_Hu_Rv | CCCAACCTGCACAGTAGCGCA |
| BLBP_Hu_Fw | GTCTGTTGTTAGCCTGGATGGAGAC | BLBP_Hu_Rv | AGTGGCGAACAGCAACCACATCA |
| BRN3a_Hu_Fw | AGCAAGCAGCCTCACTTTGC | BRN3a_Hu_Rv | CTTGAAAGGATGGCTCTTGC |
| CALB1_Hu_Fw | GGCTCACGTATTACCCACAGA | CALB1_Hu_Rv | GAAGCCACTGTGGTCAGTATCA |
| CORIN_Hu_Fw | CACAGCCAGGGTCTGGTGGAATGCAG | CORIN_Hu_Rv | GAGAGCTACCACCACATGAATCAAGG |
| DAT_Hu_Fw | TCATCGCCACATCCTCCA | DAT_Hu_Rv | ACCAGCTCACGGTCCTTCTC |
| EN1_Hu_Fw | AGCGCAGGGCACCAAATAC | EN1_Hu_Rv | GGACGATCCGAATAACGTGTG |
| EN2_Hu_Fw | GGTCTACTGTACGCGCTACTCG | EN2_Hu_Rv | CTTTGTTCGGGTTCTTCTTCTTTG |
| FN1_Hu_Fw | AAAAAGACAGACGAGCTTCCCCAACT | FN1_Hu_Rv | GGGTGACGAAAGGGGTCTTTTGA |
| FOXA2_Hu_Fw | TTCAGGCCCGGCTAACTCT | FOXA2_Hu_Rv | AGTCTCGACCCCCACTTGCT |
| GAPDH_Hu_Fw | GGTCGGAGTCAACGGATTTG | GAPDH_Hu_Rv | TCAGCCTTGACGGTGCCATG |
| GBX2_Hu_Fw | GGTAACTTCGACAAGGCGGAGG | GBX2_Hu_Rv | GGTCGTCTTCCACCTTTGACTCG |
| GFAP_Hu_Fw | GTTCTCTCGGAGTATCTGG | GFAP_Hu_Rv | GATATCCCACCTCATAAAAACC |
| GLI1_Hu_Fw | GGTCCCATCAGGGAGGAAAG | GLI1_Hu_Rv | TCGTCCAAGCTGGAGAGGTC |
| GSC_Hu_Fw | GAGGAGAAAGTGGAGGTCTGGTT | GSC_Hu_Rv | CTCTGATGAGGACCGCTTCTG |
| HES5_Hu_Fw | GCACATTTGCCTTTTGTGAA | HES5_Hu_Rv | CACACTCAGGAGCCTTTTGG |
| HOXA2_Hu_Fw | GGATGAAGGAGAAGAAGGCGG | HOXA2_Hu_Rv | CTGCCATCGGCGATTTCCAGG |
| HOXB4_Hu_Fw | GCGCAAAGTTCACGTGAGCAC | HOXB4_Hu_Rv | GGAACCAGATCTTGATCTGGCG |
| KCNJ6_Hu_Fw | GCTCGAAGCTCCTACATCACC | KCNJ6_Hu_Rv | CTCTTTGGCACTAAGGGATGG |
| LMX1A_Hu_Fw | GATCCCTTCCGACAGGGTCTC | LMX1A_Hu_Rv | GGTTTCCCACTCTGGACTGC |
| LMX1B_Hu_Fw | TGTGCAAGGGTGACTACGAGAA | LMX1B_Hu_Rv | TTCATGTCCCCATCTTCATCCT |
| LRP4_Hu_Fw | CAGGAGGTGGTAGTGGATAC | LRP4_Hu_Rv | GACGATCAAGGTTCTCCCAG |
| LRTM1_Hu_Fw | ATTGCCACTTGCTCGGTCTT | LRTM1_Hu_Rv | TCCTTTCCCTTCCAGGTGTCT |
| MAP2_Hu_Fw | GGATCAACGGAGAGCTGAC | MAP2_Hu_Rv | TCAGGACTGCTACAGCCTCA |
| MKI67_Hu_Fw | AAGCCCTCCAGCTCCTAGTC | MKI67_Hu_Rv | GCAGGTTGCCACTCTTTCTC |
| NANOG_Hu_Fw | GGCTCTGTTTTGCTATATCCCCTAA | NANOG_Hu_Rv | CATTACGATGCAGCAAATACGAGA |
| NESTIN_Hu_Fw | AACTCCCGGCTGCAAACAC | NESTIN_Hu_Rv | GGACTGGGAGCAAAGATCCA |
| Neurogenin2_Hu_Fw | CAGGCCAAAGTCACAGCAAC | Neurogenin2_Hu_Rv | CCGAGCAGCACTAACACGTC |
| NKX2.1_Hu_Fw | AACCAAGCGCATCCAATCTCAAGG | NKX2.1_Hu_Rv | TGTGCCCAGAGTGAAGTTTGGTCT |
| NKX6.1_Hu_Fw | ATCTTCTGGCCCGGAGTG | NKX6.1_Hu_Rv | TCTTCCCGTCTTTGTCCAAC |
| NR4A2_Hu_Fw | CGAAACCGAAGAGCCCACAGGA | NR4A2_Hu_Rv | GGTCATAGCCGGGTTGGAGTCG |
| OLIG2_Hu_Fw | TGCCAGGTTCTCCCCTGAGGC | OLIG2_Hu_Rv | AGTCGTCGCAGCTTTCGCAGG |
| OTX2_Hu_Fw | CATGCGAGAGGAGGTGGCAC | OTX2_Hu_Rv | CCCGAGCTGGAGATGTCTTC |
| PAX2_Hu_Fw | GACCAAAGTTCAGCAGCCTTTC | PAX2_Hu_Rv | CAGGATCCCATTGATGGAGTAG |
| PAX6_Hu_Fw | ACCCATTATCCAGATGTGTTTGCCCGAG | PAX6_Hu_Rv | ATGGTGAAGCTGGGCATAGGCGGCAG |
| PITX3_Hu_Fw | GGGCCAGGAGCACAGCGACTCA | PITX3_Hu_Rv | GCTGCCGCCGCTGCTTCTTTTT |
| POU5F1_Hu_Fw | AGACCATCTGCCGCTTTGAG | POU5F1_Hu_Rv | GCAAGGGCCGCAGCTT |
| SHH_Hu_Fw | GATGTCTGCTGCTAGTCCTCGTC | SHH_Hu_Rv | TTTGGGGTGCCTCCTCTTC |
| SIX3_Hu_Fw | CCGGAAGAGTTGTCCATGTTC | SIX3_Hu_Rv | CGACTCGTGTTTGTTGATGGC |
| SNCA_Hu_Fw | GTGTGACAGCAGTAGCCCAGAAG CAGTG | SNCA_Hu_Rv | CCTTCTTCATTCTTGCCCAACTGGTCC |
| SOX1_Hu_Fw | GCGGAGCTCGTCGCATT | SOX1_Hu_Rv | GCGGTAACAACTACAAAAAACTTGTAA |
| SOX17_Hu_Fw | CGCTTTCATGGTGTGGGCTAAGGACG | SOX17_Hu_Rv | TAGTTGGGGTGGTCCTGCATGTGCTG |
| T_Hu_Fw | ATGGAGGAACCCGGAGACA | T_Hu_Rv | TGAGGATTTGCAGGTGGACA |
| TBR1_Hu_Fw | GCCTTTCTCCTTCTATCATGCTC | TBR1_Hu_Rv | GTCAGTGGTCGAGATAATGGGA |
| TH_Hu_Fw | GCAGTTCTCGCAGGACATTG | TH_Hu_Rv | CGGCACCATAGGCCTTCA |
| TPH2_Hu_Fw | TCAGCTACTTGGCAGCTCAAC | TPH2_Hu_Rv | CTTGCCACTTTCGGTAGCAG |
| TUBB3_Hu_Fw | TGATGAGCATGGCATCGAC | TUBB3_Hu_Rv | GGCCTGAAGAGATGTCCAAA |
| VMAT2_Hu_Fw | TGGCTTTGTGCTCTTCTGG | VMAT2_Hu_Rv | CCAAAGTTCGGAGCTATGAGTC |
| WNT1_Hu_Fw | GAACTGTCCCACTGCTCCAG | WNT1_Hu_Rv | GCGGAGGTGATAGCGAAGA |
| WNT5A_Hu_Fw | TTCGCCCAGGTTGTAATTGA | WNT5A_Hu_Rv | GCAGAGAGGCTGTGCTCCTA |
| ZBTB16_Hu_Fw | CCACCCCTACGAGTGTGAGT | ZBTB16_Hu_Rv | CTCAAAGGGCTTCTCACCTG |

Supplementary Table 15. Test items evaluated in the general toxicity and tumorigenicity studies.

| **Test Items** | **Tendency** |
|---|---|
| Clinical signs | Twice a day on the day of transplantation (before administration, about 4 hours after administration); once a day on other days |
| Body weight | Once a week |
| Food consumption | Once a week |
| General physical condition and behavior: modified Irwin's method | Before transplantation, 3, and 12 months after transplantation |
| Ophthalmological examination | Before transplantation, 3, and 12 months after transplantation |
| Urinalysis:<br>color, pH, protein, glucose, ketone bodies, bilirubin, occult blood, urobilinogen | Before transplantation, 3, and 12 months after transplantation |
| Hematology:<br>red blood cell count, white blood cell count, hematocrit value, hemoglobin concentration, platelet count, mean corpuscular volume, mean corpuscular hemoglobin, mean corpuscular hemoglobin concentration, reticulocyte count, white blood cell types (eosinophil count, basophil count, neutrophil count, monocyte count, and lymphocyte count). When necessary, Wright-stained blood smears were also evaluated. | Upon necropsy or emergency euthanasia (when possible) |
| Blood chemistry:<br>aspartate transaminase, alanine transaminase, alkaline phosphatase, creatine kinase, total bilirubin, total protein, albumin, globulin, total cholesterol, triglyceride, glucose, urea nitrogen, creatinine, inorganic phosphate, calcium, sodium, potassium, chloride, protein fraction (albumin ratio, α1-globulin ratio, α2-globulin ratio, β-globulin ratio, γ-globulin ratio, and A/G ratio) | Upon necropsy or emergency euthanasia (when possible) |
| Necropsy | On the next day after the observation period, death or emergency euthanasia |
| Organ weight: | Upon necropsy<br>Target organs/tissues: lungs (including the bronchial tubes), submandibular/sublingual glands, liver (including the gallbladder), heart, kidneys, testes, epididymides, prostate gland, seminal vesicles, ovaries, uterus, brain, spleen, thymus, pituitary gland, thyroid/parathyroid glands, adrenal glands. |
| Histopathology tests:<br>H-E and immunohistochemistry staining (human cell-specific antibody, KU80; proliferation-specific antibody, KI67; dopaminergic neuron-specific antibody, TH; epithelial cell-specific antibody, pan-cytokeratin; choroid plexus-specific antibody, TTR) | Target organs/tissues: trachea, lungs (including the bronchial tubes), tongue, submandibular/sublingual glands, esophagus, stomach (forestomach, Glandular stomach), small intestine (duodenum, jejunum, ileum), large intestine (cecum, colon, rectum), pancreas, liver, gallbladder, aorta, heart, kidneys, bladders, testes, epididymides, prostate gland, seminal vesicles, ovaries, uterus, vagina, brain (site of administration, cerebrum, cerebellum, pons, medulla oblongata), spinal cord, sciatic nerves, sternum/sternal bone marrow, femur/femoral bone marrow, submandibular lymph nodes, mesenteric lymph nodes, spleen, thymus, pituitary gland, thyroid/parathyroid glands, adrenal glands, eye balls/optic nerves, lacrimal glands, Harderian glands, skeletal muscles (quadriceps muscles), mammary glands, skin (ventral), skin (dorsal), sites of gross abnormalities. |
