# Supplementary material for: Pre-clinical study of induced pluripotent stem cell-derived dopaminergic progenitor cells for Parkinson’s disease
Source: Nat Commun. 2020 Jul 6;11:3369. doi: 10.1038/s41467-020-17165-w (PMC7338530; doi:10.1038/s41467-020-17165-w)
Supplement: Supplementary file 3 — Reporting Summary [file 41467_2020_17165_MOESM3_ESM.pdf]

nature research

Corresponding author(s): JUN TAKAHASHI

Last updated by author(s): Jun 11, 2020

Reporting Summary

Nature Research wishes to improve the reproducibility of the work that we publish. This form provides structure for consistency and transparency in reporting. For further information on Nature Research policies, see Authors & Referees and the Editorial Policy Checklist .

Statistics

For all statistical analyses, confirm that the following items are present in the figure legend, table legend, main text, or Methods section.

n/a	Confirmed	
☐	☒	The exact sample size (*n*) for each experimental group/condition, given as a discrete number and unit of measurement
☐	☒	A statement on whether measurements were taken from distinct samples or whether the same sample was measured repeatedly
☐	☒	The statistical test(s) used AND whether they are one- or two-sided<br>*Only common tests should be described solely by name; describe more complex techniques in the Methods section.*
☐	☒	A description of all covariates tested
☐	☒	A description of any assumptions or corrections, such as tests of normality and adjustment for multiple comparisons
☐	☒	A full description of the statistical parameters including central tendency (e.g. means) or other basic estimates (e.g. regression coefficient) AND variation (e.g. standard deviation) or associated estimates of uncertainty (e.g. confidence intervals)
☐	☒	For null hypothesis testing, the test statistic (e.g. *F*, *t*, *r*) with confidence intervals, effect sizes, degrees of freedom and *P* value noted<br>*Give P values as exact values whenever suitable.*
☐	☒	For Bayesian analysis, information on the choice of priors and Markov chain Monte Carlo settings
☐	☒	For hierarchical and complex designs, identification of the appropriate level for tests and full reporting of outcomes
☐	☒	Estimates of effect sizes (e.g. Cohen's *d*, Pearson's *r*), indicating how they were calculated

Our web collection on statistics for biologists contains articles on many of the points above.

Software and code

Policy information about availability of computer code

Data collection	The rotation number is counted by RotoRat software version 2.01 (MED Associates, Inc., Vermont, US).
Data analysis	The statistical analyses were performed using a commercially available software package (GraphPad Prism 7, GraphPad Software Inc.)<br>Single cell gene expression was analyzed using R version 3.5.2 with Singular Analysis Toolset version 3.6.2 (Fluidigm Corp., California, US)<br>Genome variant analyses were performed with publicly available software: Genomon (1.0.1), Genomon2 (2.3.0), VarScan2 (2.4.2), Delly (0.7.3), GenomeStudio (2011.1, Illumina), PennCNV (1.0.3), MAD (1.0.1), GWAS tools (1.16.1), and ANNOVAR (2016 Feb 01).<br>DNA methylation was analyzed using GenomeStudio (2011.1, Illumina) and R (3.2.3 and 3.2.5) with BMIQ (1.5) and ggplot2 (2.0.0).<br>The genome sequencing data was analyzed by BWA MEM (ver. 0.7.15) and SAMtools (ver. 0.1.19).

For manuscripts utilizing custom algorithms or software that are central to the research but not yet described in published literature, software must be made available to editors/reviewers. We strongly encourage code deposition in a community repository (e.g. GitHub). See the Nature Research guidelines for submitting code & software for further information.

Data

Policy information about availability of data

All manuscripts must include a data availability statement. This statement should provide the following information, where applicable:

- Accession codes, unique identifiers, or web links for publicly available datasets
- A list of figures that have associated raw data
- A description of any restrictions on data availability

The authors declare that most of the data supporting the findings of this study are available within the article and its Supplementary Information files or from the corresponding author upon reasonable request, and some of the data are not publicly available due to them containing information that could compromise donor privacy.
The datasets used in this study are as follows;
HGMD Pro 2016.4: https://digitalinsights.qiagen.com/products-overview/clinical-insights-portfolio/human-gene-mutation-database/

http://www.hgmd.cf.ac.uk/ac/index.php
COSMIC 83: https://cancer.sanger.ac.uk/cosmic
Shibata's gene list: http://www.pmda.go.jp/files/000152599.pdf
refGene, genomicSuperDups, snp131, esp6500siv2_all, 1000g2015aug_all were downloaded through ANNOVAR as humandb 20160720: https://doc-openbio.readthedocs.io/projects/annovar/en/latest/

Field-specific reporting

Please select the one below that is the best fit for your research. If you are not sure, read the appropriate sections before making your selection.

☒ Life sciences ☐ Behavioural & social sciences ☐ Ecological, evolutionary & environmental sciences

For a reference copy of the document with all sections, see nature.com/documents/nr-reporting-summary-flat.pdf

Life sciences study design

All studies must disclose on these points even when the disclosure is negative.

Sample size	No sample size calculations are done. In NOG mice study, at least 20 animals are planned to be evaluated histologically. Considering the reduciton of animal number caused by natural death during life-long observation period, 80 animals are used for transplantation. In efficacy study, 20 rats are screened by methamphetamine-induced rotational behavior, and 16 rats are used for transplantation.
Data exclusions	In efficacy study, 2 dead rats during the observation are excluded.
Replication	In all experiments, we used multiple samples and the sample numbers are presented and all the data is reproduced.
Randomization	The 16 rats were assigned into two groups (saline and sample group) without randomization so that the average rotation scores in each group was similar. In other animal studies, animals were assigned randomly.
Blinding	In efficacy study using rat, blinding is not relevant because methamphetamine-induced rotation behavior is measured by rotometer automatically. In other experiments animals and samples are evaluated in a blinded manner where possible.

Reporting for specific materials, systems and methods

We require information from authors about some types of materials, experimental systems and methods used in many studies. Here, indicate whether each material, system or method listed is relevant to your study. If you are not sure if a list item applies to your research, read the appropriate section before selecting a response.

Materials & experimental systems

n/a	Involved in the study	
☐	☒	Antibodies
☐	☒	Eukaryotic cell lines
☒	☐	Palaeontology
☐	☒	Animals and other organisms
☐	☒	Human research participants
☒	☐	Clinical data

Methods

n/a	Involved in the study	
☒	☐	ChIP-seq
☐	☒	Flow cytometry
☐	☒	MRI-based neuroimaging

Antibodies

Antibodies used	Antibodies are described in Supplementary Table 11.
Validation	Antibodies are used under validation using adequate negative and positive control samples.<br>We confirmed the staining by using animal samples when possible, or we rely on the stained samples presented in manufacturer's website.

Eukaryotic cell lines

Policy information about cell lines

Cell line source(s)	iPSCs are derived from human peripheral blood cells, donated by healthy volunteer.<br>QHJI01s04 and 201B7 are provided by Center for iPS cell Research and Application (CiRA), Kyoto University, Japan.<br>MCB003 are provided by Sumitomo Dainippon Pharma (Tokyo, Japan).<br>HeLa cells are provided by Shin Nippon Biochemedical Laboratories (Kagoshima, Japan).

Authentication | The STR (short tandem repeat) pattern of iPSCs coincide with that of donor cells. None of other cell lines (201B7, HeLa) were authenticated.

Mycoplasma contamination | All cell lines are negative for mycoplasma contamination.

Commonly misidentified lines (See ICLAC register) | Nothing.

Animals and other organisms

Policy information about studies involving animals; ARRIVE guidelines recommended for reporting animal research

Laboratory animals | 7-8-week-old male and female NOG mice (NOD.Cg-Prkdcscid Il2rgtm1Sug/Jic, CLEA, Japan), adult (8-9-week-old) male nude rats (F344/NJcl-rnu/rnu, CLEA, Japan), and adult (4-7-year-old) male cynomolgus monkeys (Macaca fascicularis, Shin Nippon Biomedical Labratories, Kagoshima, Japan) were used for this study.

Wild animals | The study did not involve wild animals.

Field-collected samples | The study did not involve samples collected from the field.

Ethics oversight | We follow the guidelines of animal experiments of Kyoto University and Shin Nippon Biomedical Laboratories. Animal experiments are certified by an ethical committee at Kyoto University and Shin Nippon Biomedical Laboratories (Kagoshima, Japan).

Note that full information on the approval of the study protocol must also be provided in the manuscript.

Human research participants

Policy information about studies involving human research participants

Population characteristics | A volunteer donor was recruited.

Recruitment | We obtained information regarding people who have already had their HLA type examined, and ask those who are HLA homozygous to take part in the research project of their own free will.
Details of donor recruitment is mentioned in ref#8.

Ethics oversight | The study protocol is approved by the ethical committee of Kyoto University, Kyoto, Japan.

Note that full information on the approval of the study protocol must also be provided in the manuscript.

Flow Cytometry

Plots

Confirm that:

- [ ] The axis labels state the marker and fluorochrome used (e.g. CD4-FITC).
- [ ] The axis scales are clearly visible. Include numbers along axes only for bottom left plot of group (a 'group' is an analysis of identical markers).
- [ ] All plots are contour plots with outliers or pseudocolor plots.
- [x] A numerical value for number of cells or percentage (with statistics) is provided.

Methodology

Sample preparation | Cultivated cells are harvested and dissociated to single cells by enzymatic reaction and mechanical pipetting.

Instrument | Canto II, FACSAria, Influx cell sorter (BD Biosciences)

Software | FACSDiva and BD FACS Sortware (BD)

Cell population abundance | Sorted cells are reanalyzed immediately after sorting and confirmed that the purity is over 90% of live cells.

Gating strategy | The positive gate was set so that less than 0.1% of cells were positive in unstained samples or samples stained by an isotype control antibody.

- [x] Tick this box to confirm that a figure exemplifying the gating strategy is provided in the Supplementary Information.

Magnetic resonance imaging

Experimental design

Design type: Resting state, Structural MRI imaging

Design specifications: Not applicable, fMRI was not a part of this study.

Behavioral performance measures: Not applicable, fMRI was not a part of this study.

Acquisition

Imaging type(s): structual imaging

Field strength: 3-Tesla

Sequence & imaging parameters: T2-weighted image, T2 SPACE sequence (TR=2500 ms, TE=301 ms, base resolution=128, FOV=102 mm, Slice thickness=0.8 mm, Turbo factor=77, Slice turbo factor=2)

Area of acquisition: A whole brain scan

Diffusion MRI: ☐ Used ☒ Not used

Preprocessing

Preprocessing software: Functional Magnetic Resonance Images of Brain (FMRIB) software libraries

Normalization: Normalized with structurally to the standardized MNI space of macaca fascicularis.

Normalization template: The standardized MNI space of macaca fascicularis

Noise and artifact removal: Noise and artifact was not removed.

Volume censoring: No volumes censoring was performed.

Statistical modeling & inference

Model type and settings: The grafted cells are confirmed in putamen of the brain after cell transplantation.

Effect(s) tested: Not applicable, fMRI was not a part of this study.

Specify type of analysis: ☐ Whole brain ☒ ROI-based ☐ Both

Anatomical location(s): ROIs were determined the graft region after transplantation.

Statistic type for inference (See Eklund et al. 2016): Not applicable, fMRI was not a part of this study.

Correction: Not applicable, fMRI was not a part of this study.

Models & analysis

n/a	Involved in the study
☒	☐ Functional and/or effective connectivity
☒	☐ Graph analysis
☒	☐ Multivariate modeling or predictive analysis
