# Supplementary material for: Pre-clinical study of induced pluripotent stem cell-derived dopaminergic progenitor cells for Parkinson’s disease
Source: Nat Commun. 2020 Jul 6;11:3369. doi: 10.1038/s41467-020-17165-w (PMC7338530; doi:10.1038/s41467-020-17165-w)
Supplement: Supplementary file 4 — Description of Additional Supplementary Files [file 41467_2020_17165_MOESM4_ESM.pdf]

Description of Additional Supplementary Files

Title: Supplementary Data 1.

Description: Ct value of single cell-derived qPCR, related to Figure 2c
